# Supplementary figures and images for: Mutant mice lacking alternatively spliced p53 isoforms unveil Ackr4 as a male-specific prognostic factor in Myc-driven B-cell lymphomas
Source: eLife. 2024 Sep 19;13:RP92774. doi: 10.7554/eLife.92774 (PMC11412721; doi:10.7554/eLife.92774)

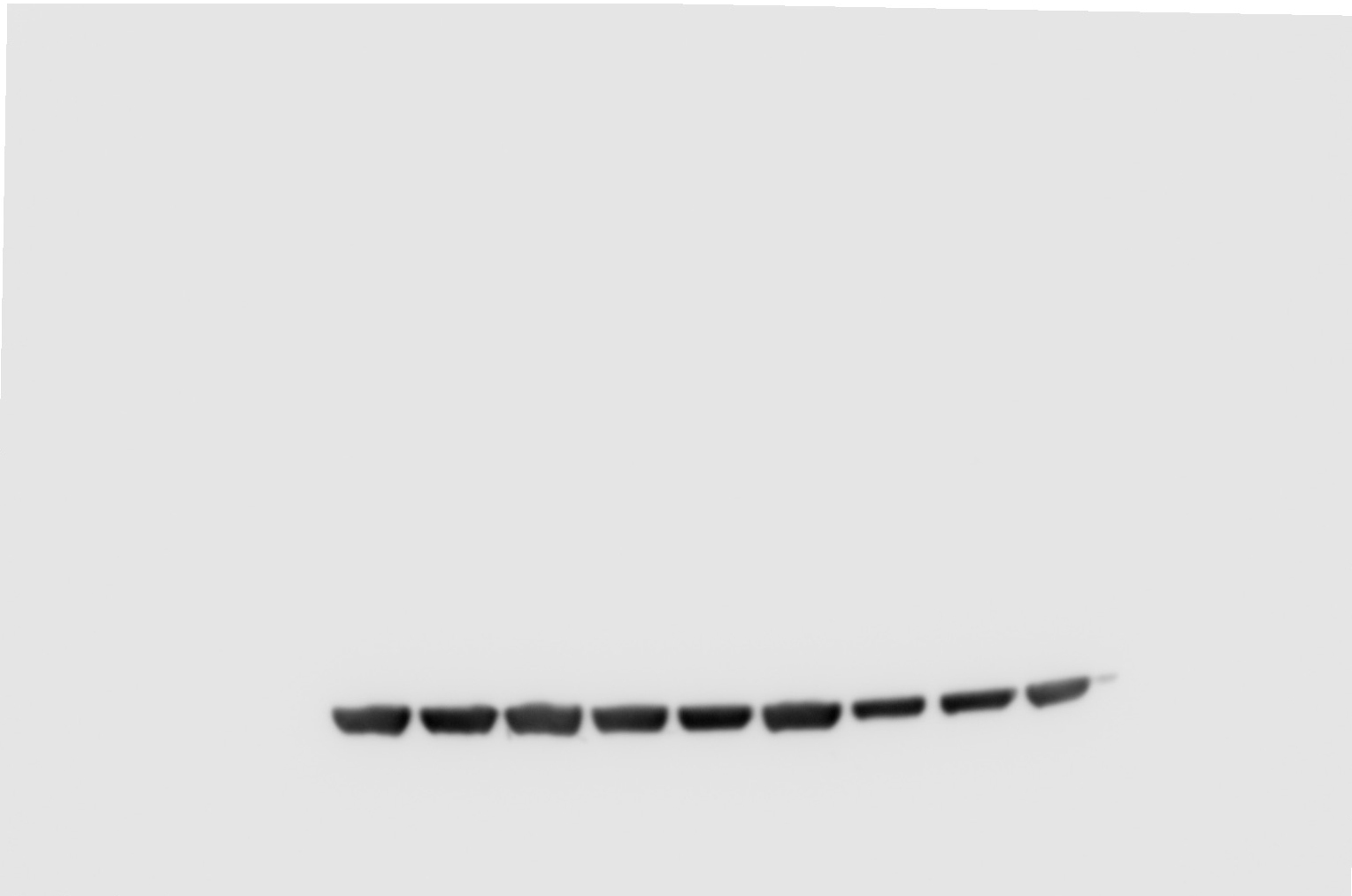

Supplement: Figure 1—source data 1. [file elife-92774-fig1-data1.zip › Fajac-Fig1-Sourcedata1-actin-raw.jpg]

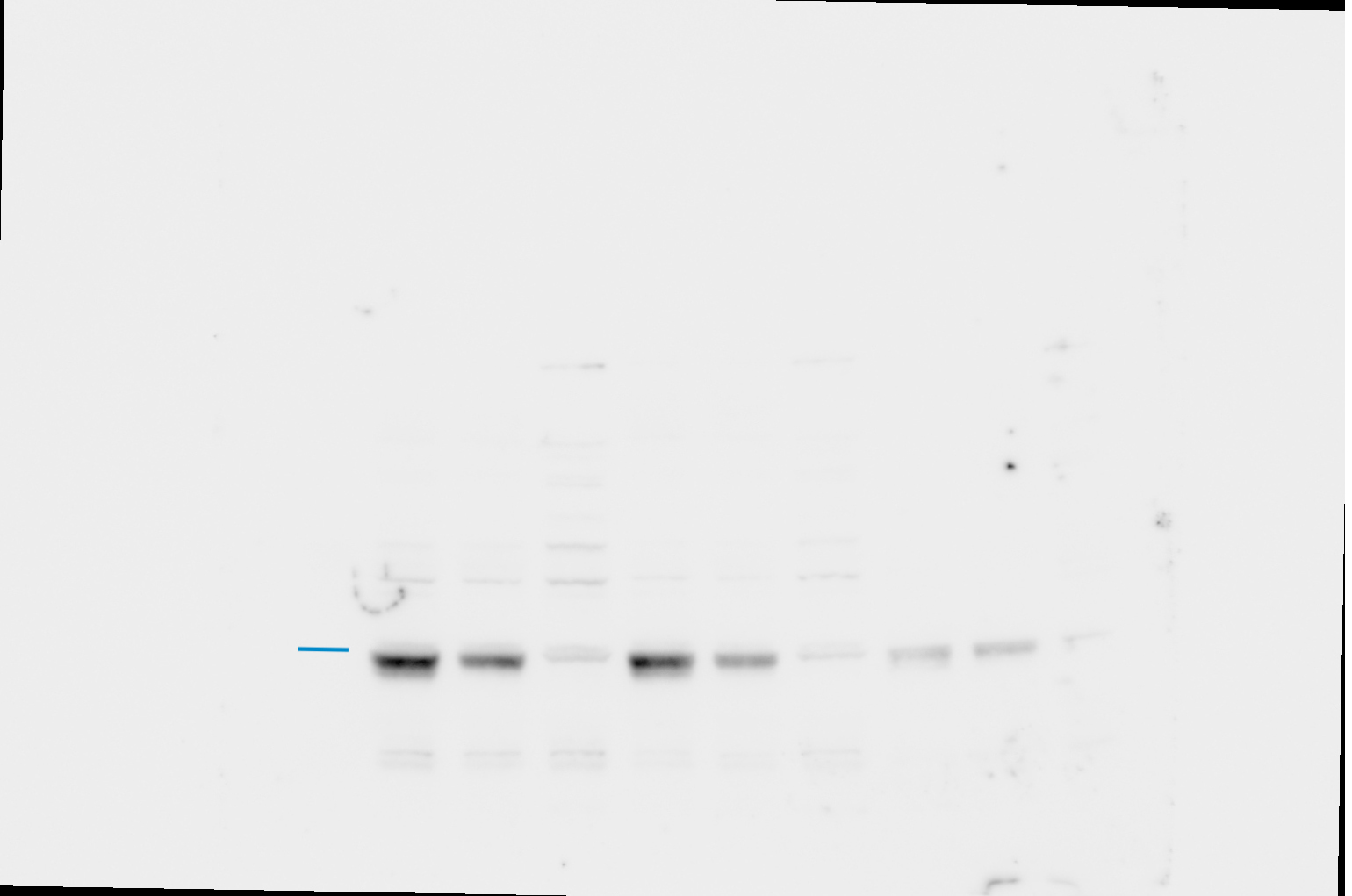

Supplement: Figure 1—source data 1. [file elife-92774-fig1-data1.zip › Fajac-Fig1-Sourcedata1-p53-raw.jpg]

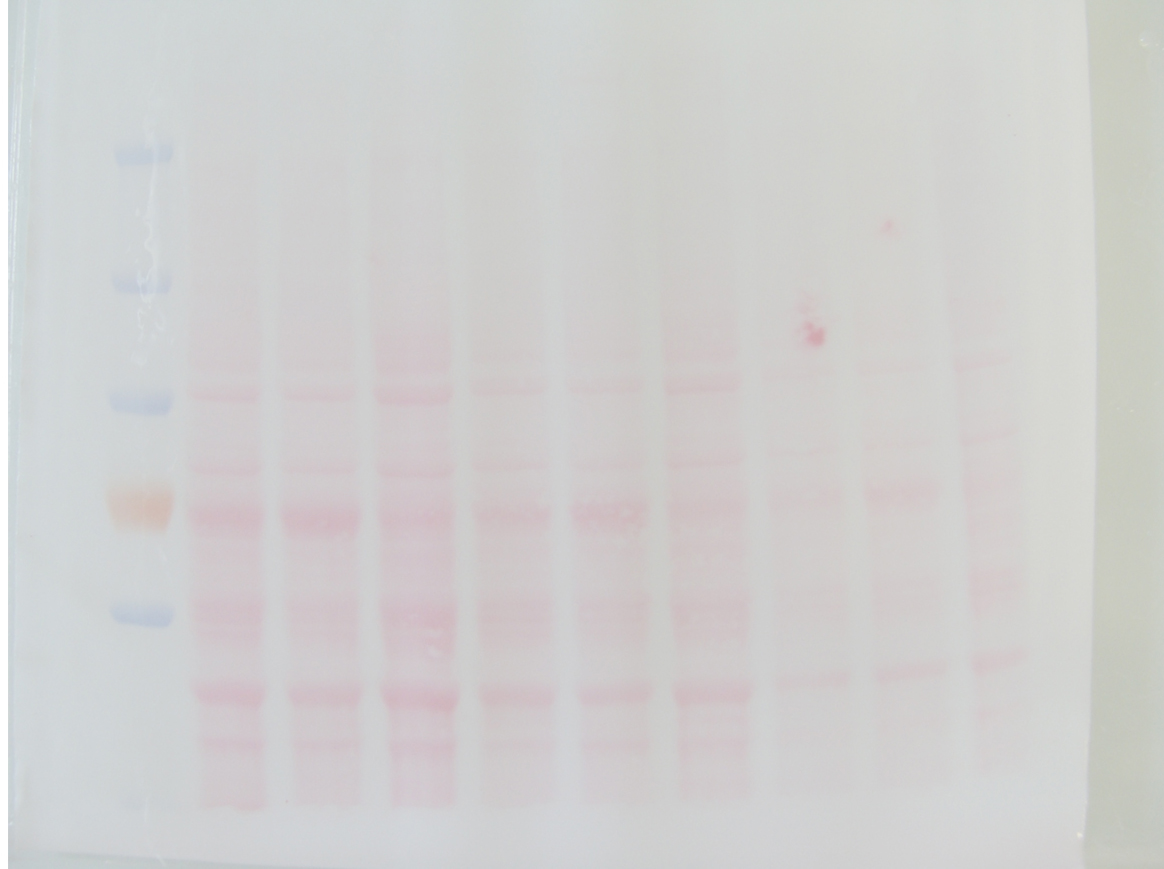

Supplement: Figure 1—source data 1. [file elife-92774-fig1-data1.zip › Fajac-Fig1-Sourcedata1-ponceau-raw.jpg]

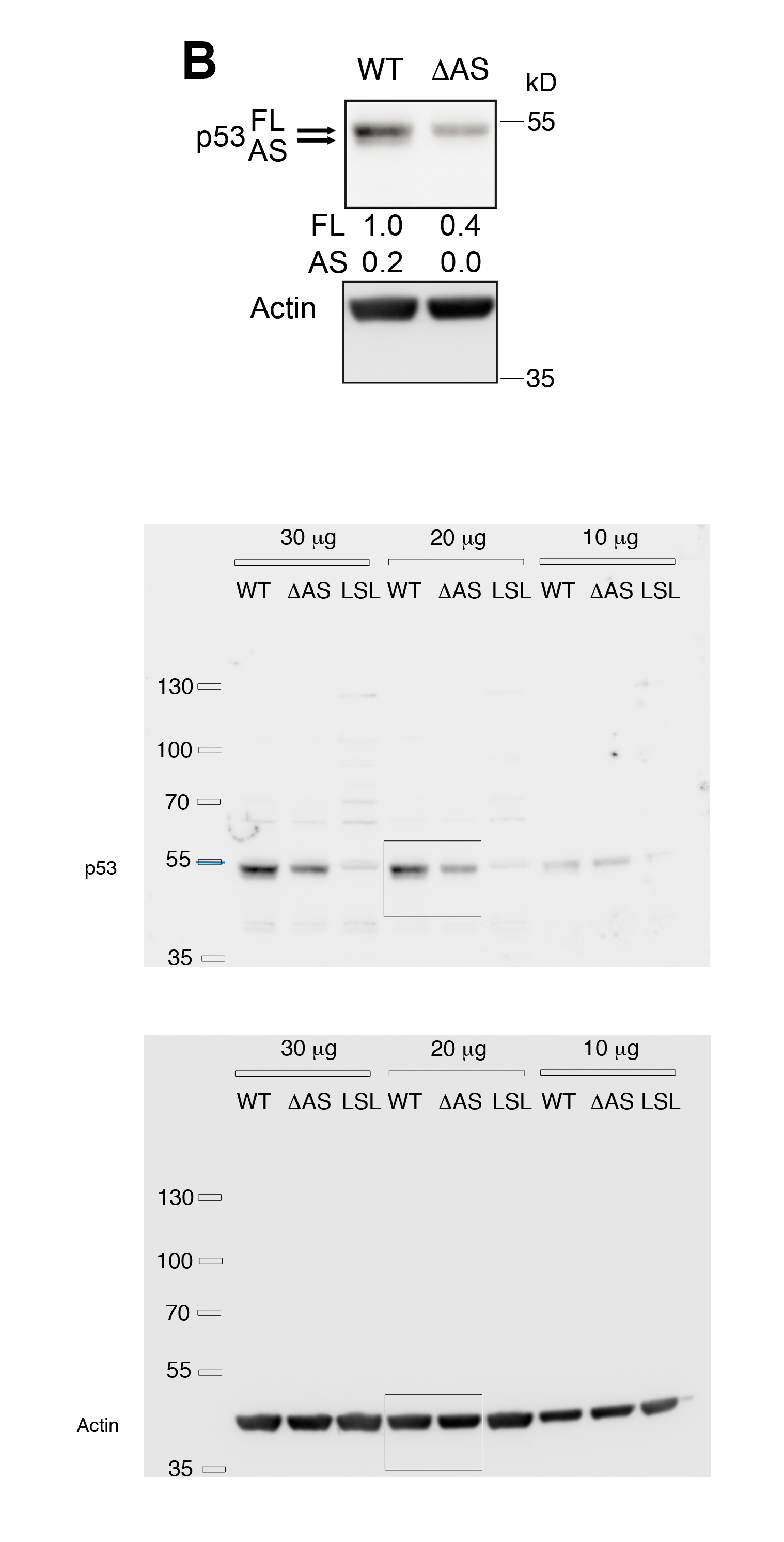

Supplement: Figure 1—source data 2. [file elife-92774-fig1-data2.jpg]

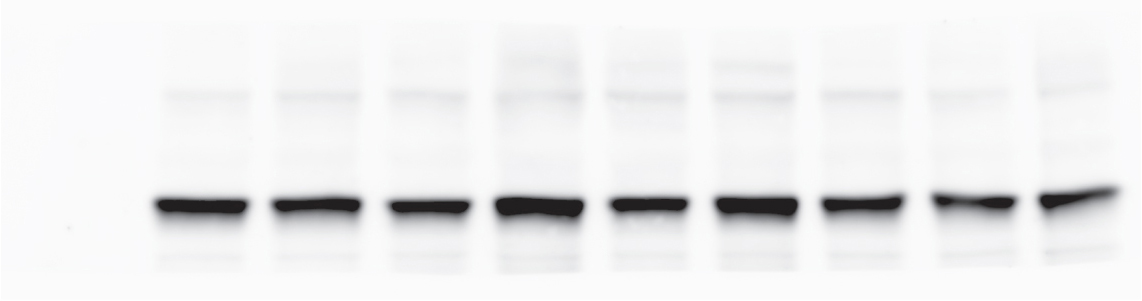

Supplement: Figure 2—source data 1. [file elife-92774-fig2-data1.zip › Fajac-Fig2C-sourcedata1-myc-raw.jpg]

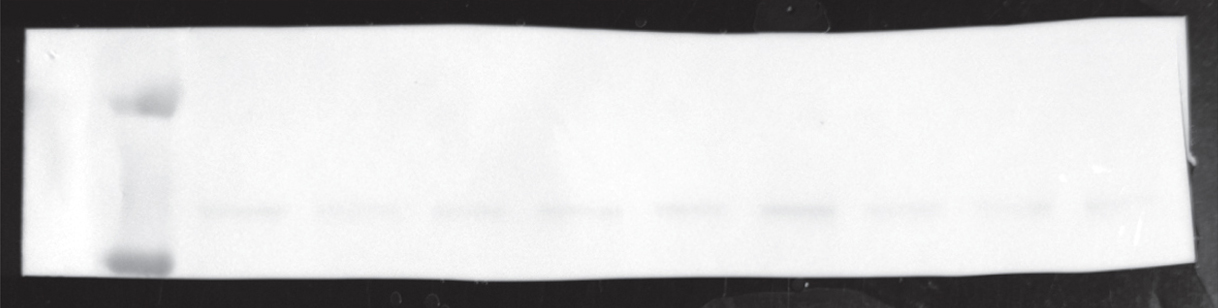

Supplement: Figure 2—source data 1. [file elife-92774-fig2-data1.zip › Fajac-Fig2C-sourcedata1-MW-raw.jpg]

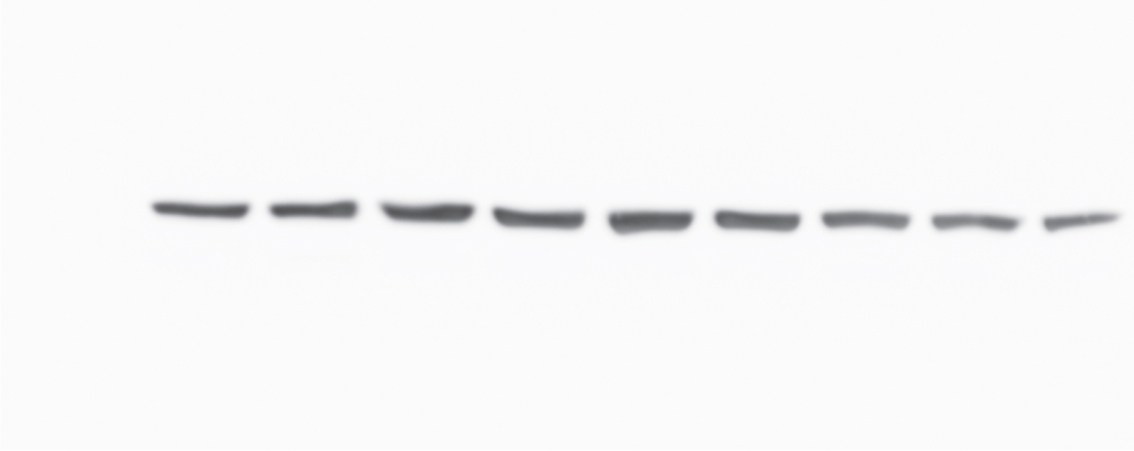

Supplement: Figure 2—source data 1. [file elife-92774-fig2-data1.zip › Fajac-Fig2C-sourcedata1-actin-raw.jpg]

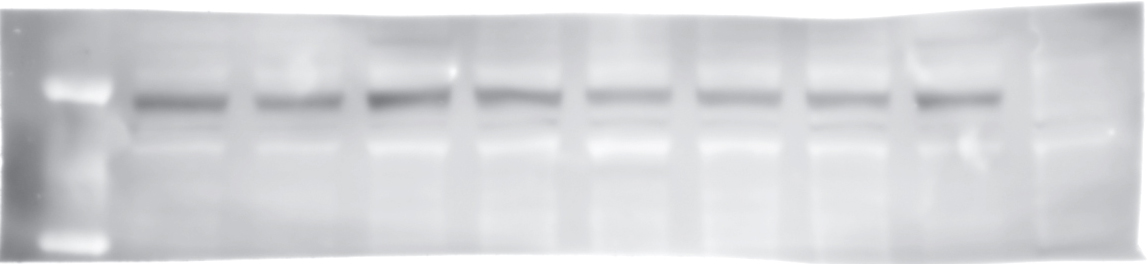

Supplement: Figure 2—source data 2. [file elife-92774-fig2-data2.zip › Fajac-Fig2D-sourcedata1-p53-raw.jpg]

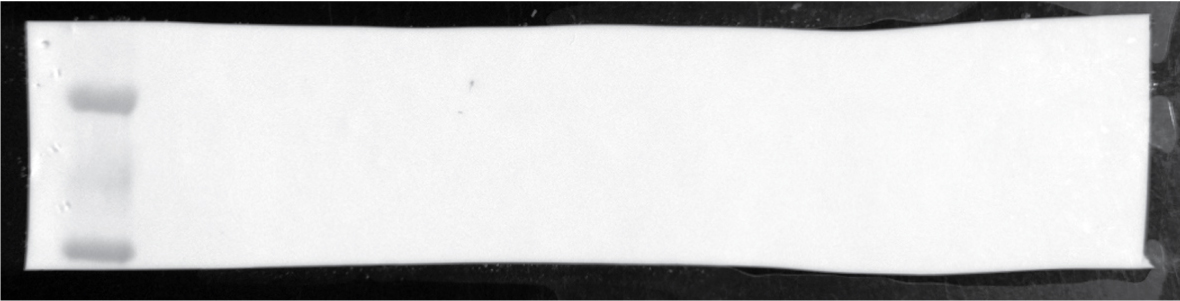

Supplement: Figure 2—source data 2. [file elife-92774-fig2-data2.zip › Fajac-Fig2D-sourcedata1-MW-raw.jpg]

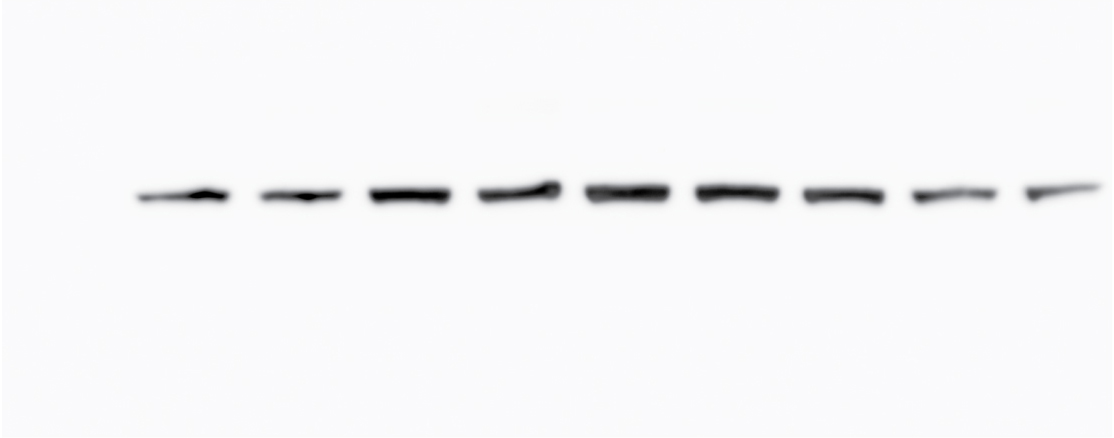

Supplement: Figure 2—source data 2. [file elife-92774-fig2-data2.zip › Fajac-Fig2D-sourcedata1-actin-raw.jpg]

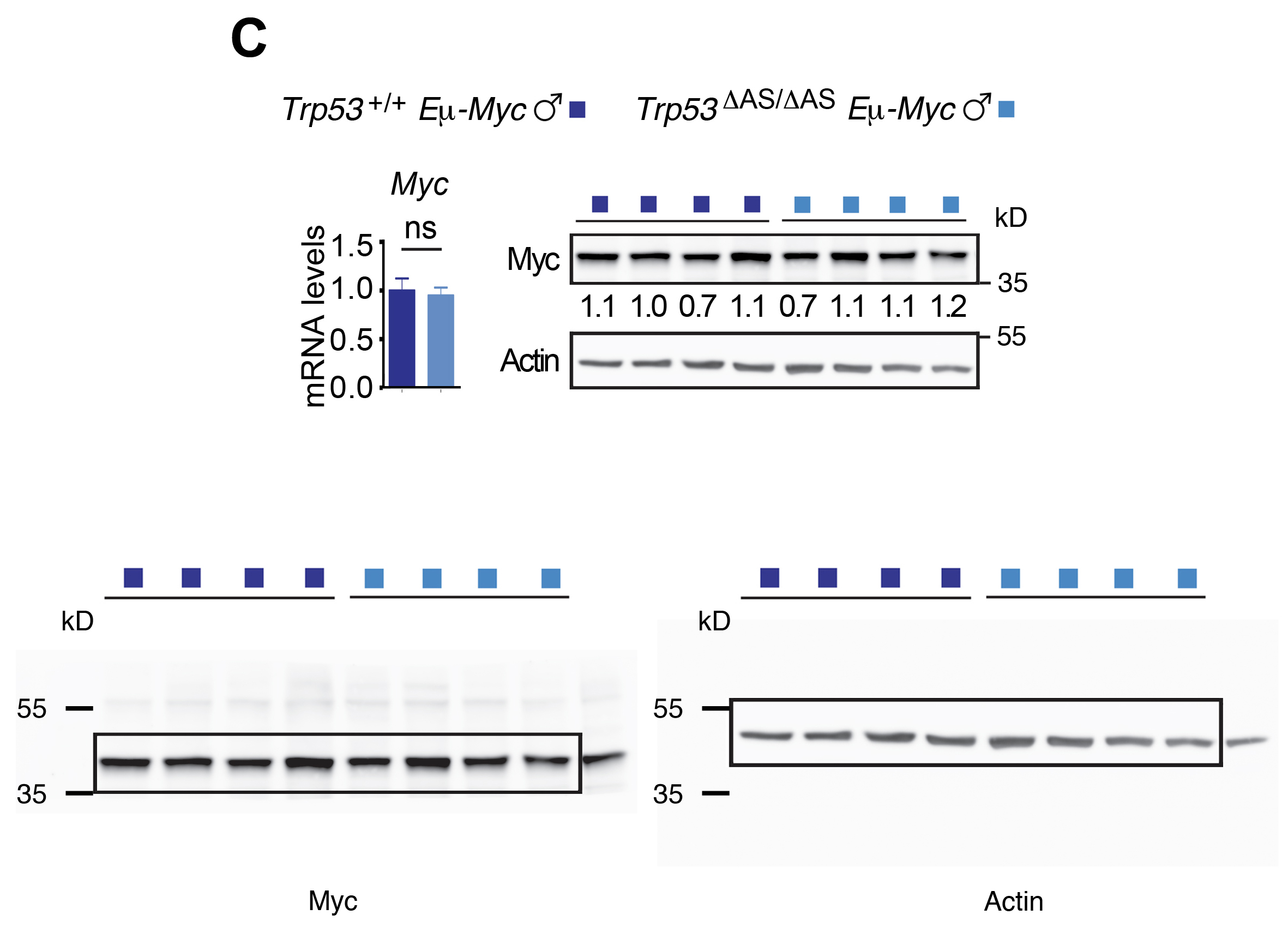

Supplement: Figure 2—source data 3. [file elife-92774-fig2-data3.jpg]

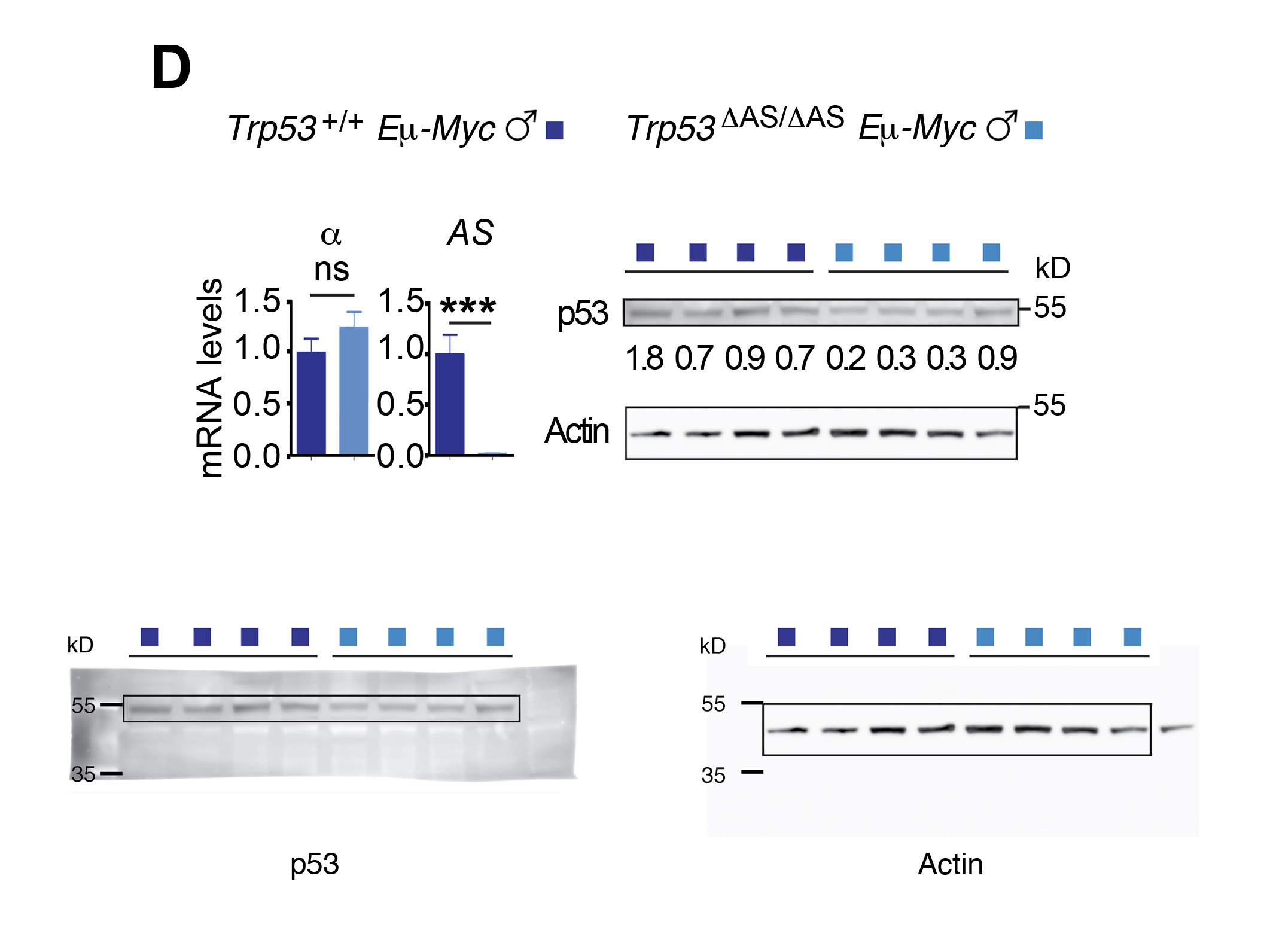

Supplement: Figure 2—source data 4. [file elife-92774-fig2-data4.jpg]

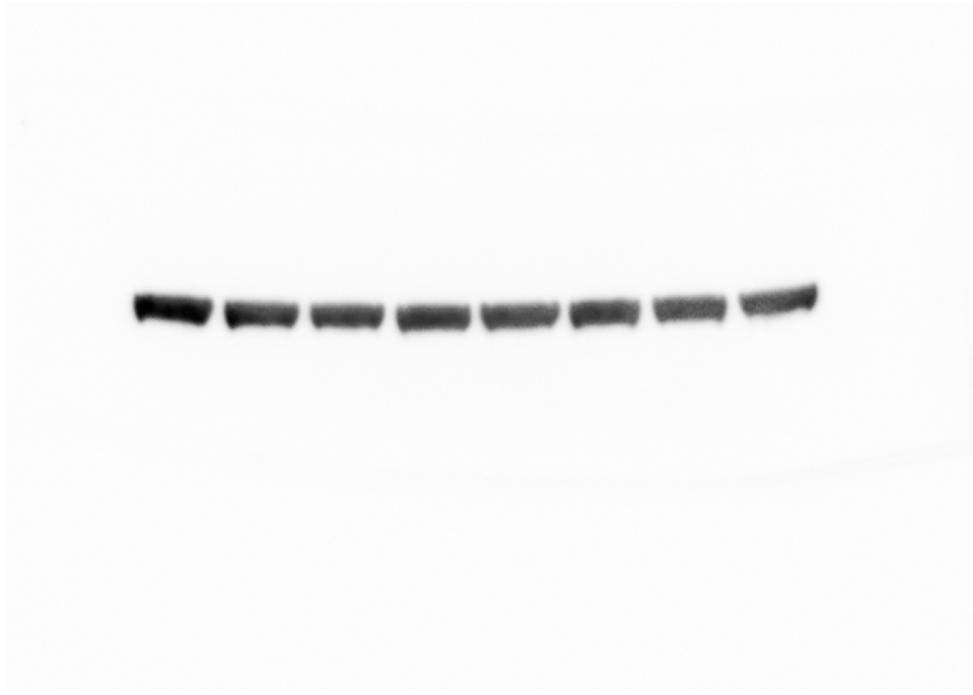

Supplement: Figure 3—figure supplement 1—source data 1. [file elife-92774-fig3-figsupp1-data1.zip › Fajac-Fig3figsupp1-Sourcedata1-actin-raw.jpg]

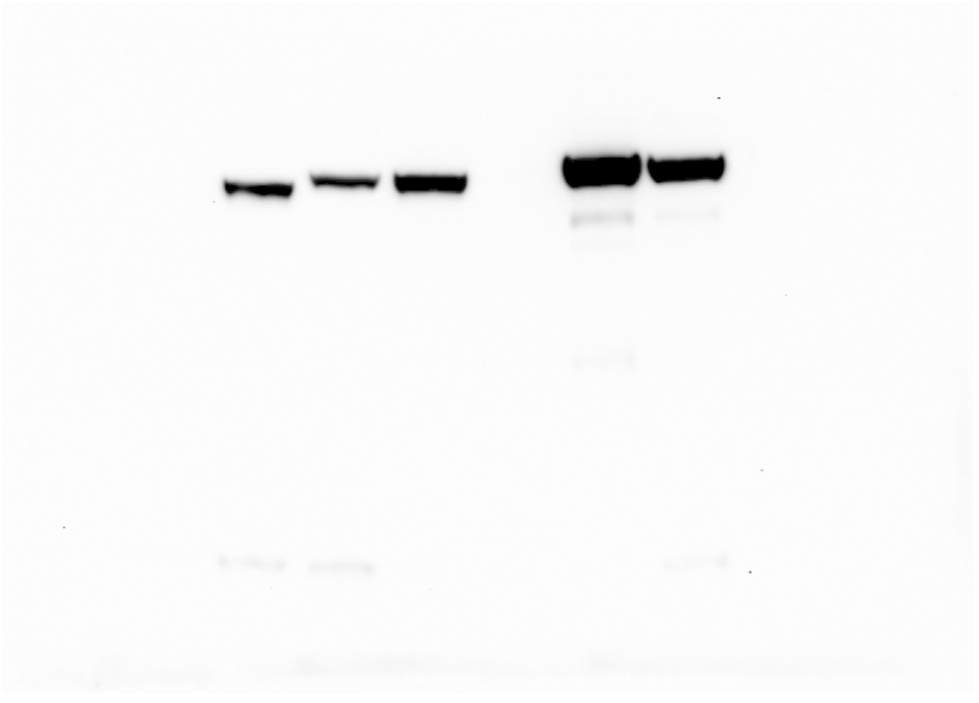

Supplement: Figure 3—figure supplement 1—source data 1. [file elife-92774-fig3-figsupp1-data1.zip › Fajac-Fig3figsupp1-Sourcedata1-p53p21lowexpo-raw.jpg]

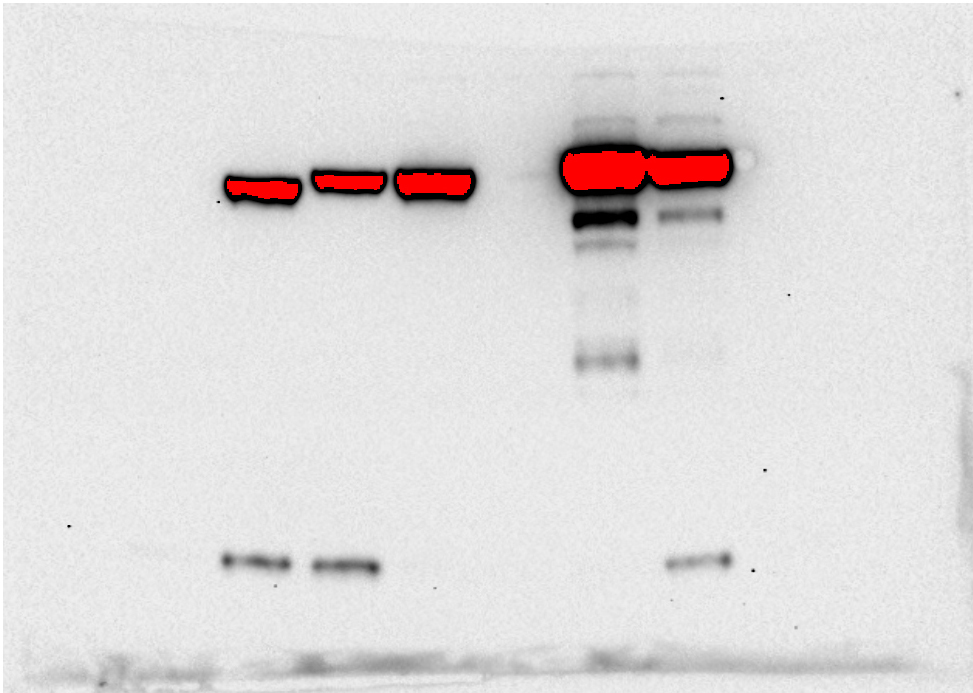

Supplement: Figure 3—figure supplement 1—source data 1. [file elife-92774-fig3-figsupp1-data1.zip › Fajac-Fig3figsupp1-Sourcedata1-p53p21highexpo-raw.jpg]

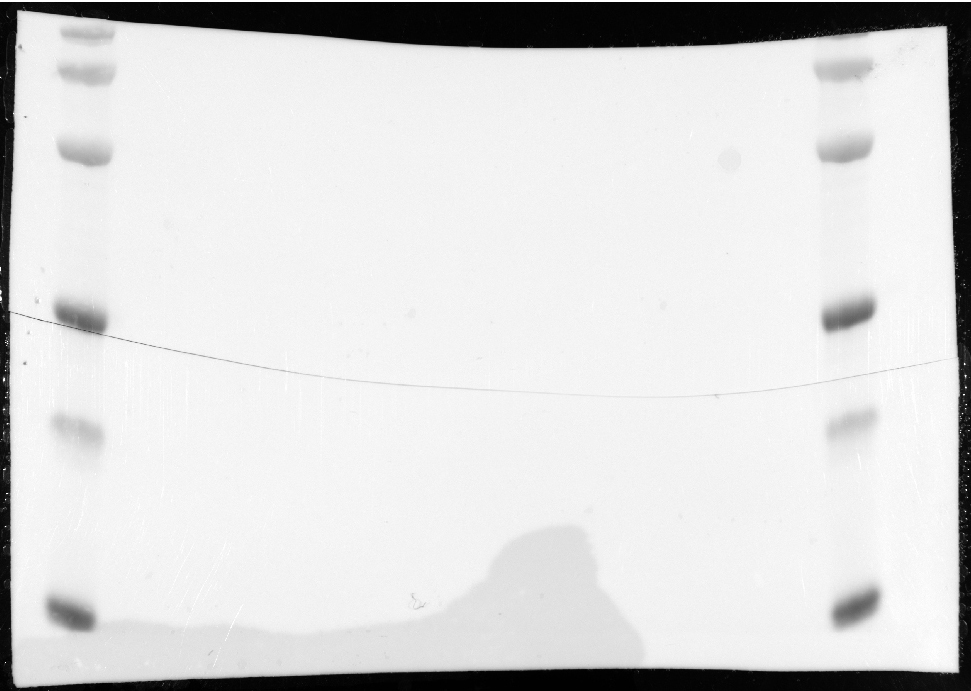

Supplement: Figure 3—figure supplement 1—source data 1. [file elife-92774-fig3-figsupp1-data1.zip › Fajac-Fig3figsupp1-Sourcedata1-MW-raw.jpg]

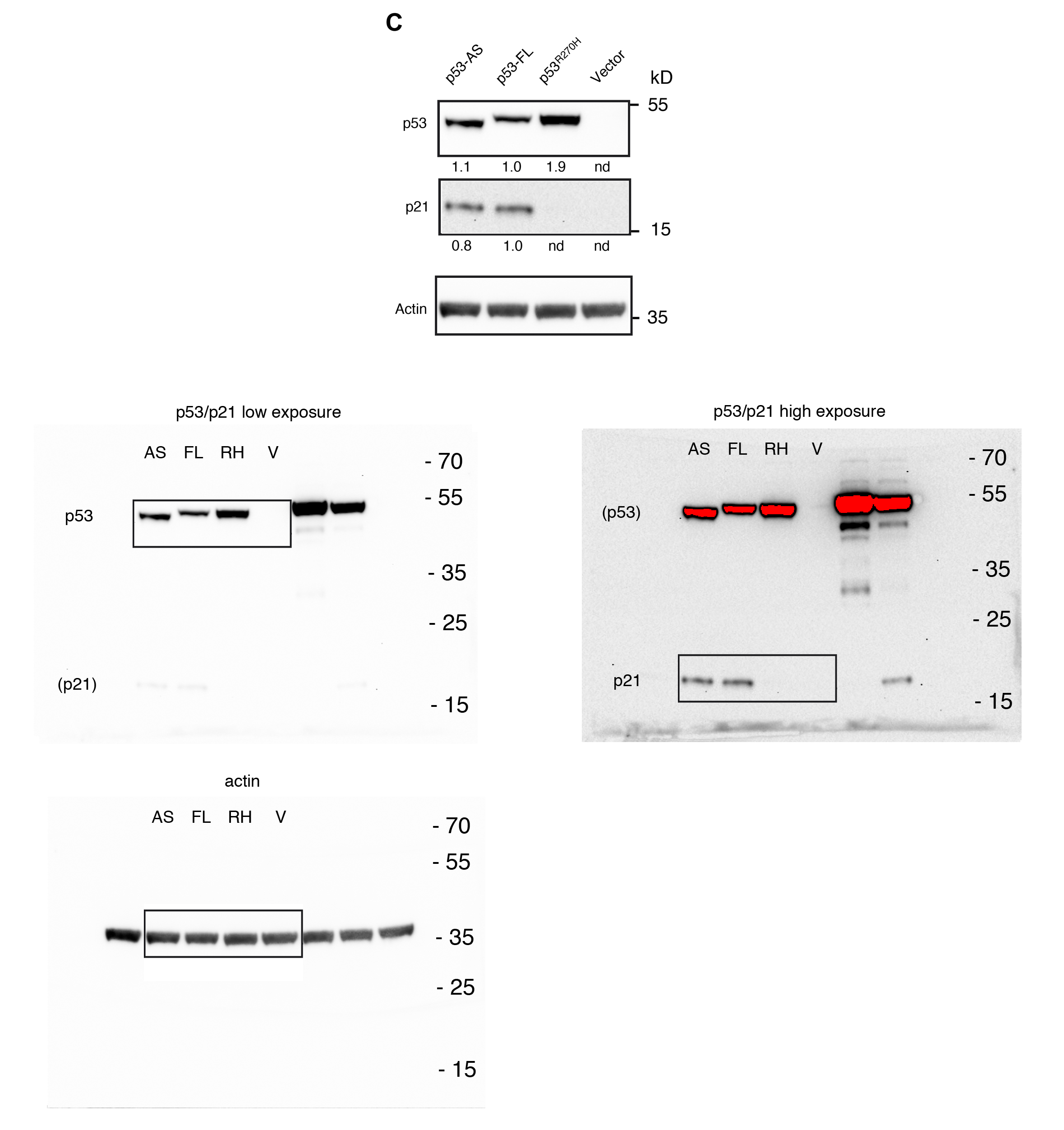

Supplement: Figure 3—figure supplement 1—source data 2. [file elife-92774-fig3-figsupp1-data2.jpg]
