# Supplementary material for: Mutant mice lacking alternatively spliced p53 isoforms unveil Ackr4 as a male-specific prognostic factor in Myc-driven B-cell lymphomas
Source: eLife. 2024 Sep 19;13:RP92774. doi: 10.7554/eLife.92774 (PMC11412721; doi:10.7554/eLife.92774)
Supplement: Supplementary file 1. — Read numbers for the indicated genes, obtained by Bulk RNA-seq from the spleens of three Trp53+/+ Eμ-Myc (WT_Myc) and four Trp53ΔAS/ΔAS Eμ-Myc (ΔAS_Myc) male mice. [file elife-92774-supp1.docx]

| **Gene** | **WT_Myc_1** | **WT_Myc_2** | **WT_Myc_3** | **ΔAS_Myc_1** | **ΔAS_Myc_2** | **ΔAS Myc_3** | **ΔAS _Myc_4** |
| --- | --- | --- | --- | --- | --- | --- | --- |
| *Ackr4* | 267.610698766789 | 187.913317967838 | 103.698438238889 | 80.2839670298248 | 58.3264519788058 | 44.2019721676723 | 58.626719832054 |
| *Cdkn1a* | 1172.3897279307 | 1179.72136964871 | 1227.565295909 | 1248.51861547663 | 1018.37985154995 | 800.288338193645 | 1079.90417930643 |
| *Mdm2* | 4419.00743604647 | 4820.24950903391 | 5133.53980290714 | 4756.31040570283 | 4240.33305885918 | 4132.88439767736 | 5521.46447378284 |
